# Kinome-Wide Screening Identifies FAK as a Novel Post-Translational Regulator of PD-L1 Stability and Immune Evasion in Triple-Negative Breast Cancer

**DOI:** 10.3390/ijms262010108

**Published:** 2025-10-17

**Authors:** Asia-Lily Boyd, Prem Khanal, Tynan Kelly, Anni Ge, Yawei Hao, Xiaolong Yang

**Affiliations:** Department of Pathology and Molecular Medicine, Queen’s University, Kingston, ON K7L 3N6, Canada; 18aljb@queensu.ca (A.-L.B.); khanalpr@yahoo.com (P.K.); 18tnpk@queensu.ca (T.K.); 21ag87@queensu.ca (A.G.); yh5@queensu.ca (Y.H.)

**Keywords:** breast cancer, PD-L1 stability, focal adhesion kinase, immunotherapy, immune evasion, kinome screen, kinase inhibitors

## Abstract

Triple-negative breast cancer (TNBC) is an aggressive subtype characterized by limited treatment options and poor prognosis. Although immune checkpoint inhibitors targeting the PD-1/PD-L1 axis have shown clinical promise, many TNBC patients exhibit resistance or limited response, underscoring the need to understand regulatory mechanisms of PD-L1 expression. Here, we performed a kinome-wide inhibitor screen using a HEK293A cell line stably expressing a NanoLuc-tagged PD-L1 construct lacking its endogenous promoter, to identify post-translational regulators of PD-L1 stability. We identified focal adhesion kinase (FAK) as a novel modulator of PD-L1. FAK inhibition significantly decreased PD-L1 levels in HEK293A cells but paradoxically increased PD-L1 expression in TNBC cell lines. Mechanistically, FAK directly interacts with PD-L1 to modulate its stability independently of its kinase activity. Functionally, FAK inhibition enhanced membrane PD-L1 expression and reduced T-cell-mediated cancer cell killing, suggesting increased immune evasion. These findings reveal a novel role for FAK in immune modulation and suggest that combining FAK inhibitors with PD-L1 blockade may offer a promising strategy for TNBC treatment.

## 1. Introduction

Cancer immunotherapy has revolutionized the treatment landscape for various malignancies, offering new hope for patients with previously intractable diseases. Among the most promising approaches in this field is the targeting of immune checkpoint molecules, particularly the programmed death-ligand 1 (PD-L1) and its receptor, programmed cell death protein 1 (PD-1). The PD-1/PD-L1 axis plays a crucial role in maintaining immune homeostasis and preventing autoimmunity under normal physiological conditions. PD-L1 is overexpressed in cancer cells, which enhances their tumorigenic potential intrinsically (immune-independent) [1,2,3,4] or through immune evasion of immune surveillance via PD-1-mediated T-cell inactivation [5,6,7,8]. Therefore, blocking inhibitory signaling by PD-1/PD-L1 with antibodies can suppress tumor growth by directly inhibiting tumor cell proliferation or reactivating cytotoxic T-cell behavior [6,9,10]. Many PD-1/PD-L1 blocking antibodies have been approved by the FDA for the treatment of multiple cancer types such as melanoma and lung cancer [10,11,12]. Over 2000 PD-1/PD-L1 blockade clinical trials in various cancers are ongoing globally, and many patients are displaying impressive responses [13]. Therefore, anti-PD-1/PD-L1 immunotherapy is currently one of the most promising cancer therapies. Although durable responses have been achieved in melanoma and non-small cell lung cancer, objective response rates in breast cancer—particularly in TNBC—remain modest [14,15,16]. This underscores the need for a deeper understanding of the molecular mechanisms governing PD-L1 expression and regulation in cancer cells.

PD-L1 expression is regulated by multiple mechanism including cytokine signaling (e.g., IFN-γ/JAK-STAT), oncogenic pathways (PI3K/AKT/mTOR, Hippo/YAP/TAZ, MAPK) and post-translational modifications that govern protein stability and trafficking. Kinases orchestrate many of these processes and are highly druggable, yet only a few kinases, including GSK3β, AMPK, ROCK and CK2, have been shown to act directly on PD-L1 [17,18]. Although targeting PD-L1 or kinases involved in cancer alone has some clinical benefit in certain types of cancers, intrinsic or acquired resistance often arises after treatment. Recent studies strongly suggest that combined treatment of cancer with kinase inhibitors and anti-PD-L1 immunotherapy may overcome resistance of cancers to single-drug therapy [19,20,21,22,23,24,25]. Therefore, identification of novel kinases interacting with PD-L1 may provide novel therapeutic strategies. We therefore undertook an unbiased kinome-wide screen to map new kinase regulators of PD-L1.

Using a HEK293A line that constitutively expresses NanoLuc (NL)-tagged PD-L1 devoid of its native promoter, we interrogated >500 kinases with selective inhibitors. This approach bypasses transcriptional inputs and focuses on post-translational control. The screen highlighted FAK as a strong hit. FAK integrates integrin and growth-factor signaling to control adhesion, migration and survival [26]. FAK is frequently overexpressed in cancers including TNBC and is currently being tested in clinical trials [20,21]. Yet its role in tumor immune evasion remains poorly defined. Here we delineate a previously unrecognized FAK–PD-L1 axis with therapeutic implications for TNBC.

## 2. Results

### 2.1. Establishment of a Cell Line Stably Expressing NanoLuc Tagged PD-L1

**Figure 1:**
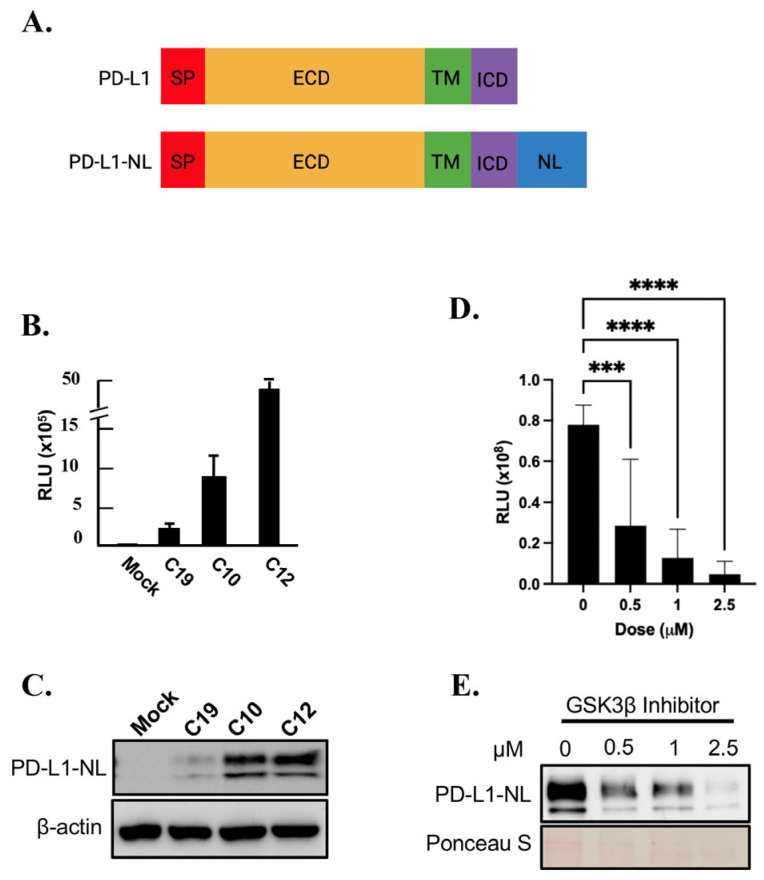
Establishing and characterization of HEK293A-PD-L1-NL stable cell line for monitoring PD-L1 stability. (**A**) Schematic representation of PD-L1 and PD-L1-NL construct. NL, NanoLuc; SP, signal peptide; ECD, extracellular domain; TM, transmembrane domain; ICD, intracellular domain. (**B**) Luciferase activity of HEK293A clones (C19, C10, C12) expressing different levels of PD-L1-NL. (**C**) Western blot analysis of PD-L1-NL expression in different clones (C19, C10, C12) using anti-PD-L1 antibody. (**D**,**E**) Dose-dependent reduction in PD-L1 stability by GSK3β inhibitor in HEK293A-PD-L1-NL cells. HEK293A-PD-L1-NL cells were treated at an increasing concentration (0, 0.5, 1.0, 2.5 µM) of GSK3β inhibitor for 1 day, followed by luciferase assays (**D**) and Western blot analysis (**E**). Statistical analysis for luciferase assays (**D**): ***, *p* < 0.001; ****, *p* < 0.0001. Ponceau S staining of proteins on membrane was used as protein loading control (**E**).

To establish a stable cell line expressing a reporter for PD-L1 levels, HEK293A cells were transfected with a PD-L1-NanoLuc fusion (PD-L1-NL; Figure 1A) construct driven by the CMV promoter, followed by hygromycin resistance screening selection. Single HEK293A clones expressing PD-L1-NL were isolated via hygromycin selection, expanded and subjected to luciferase assay and Western blot analysis. As shown in Figure 1B,C, the luciferase activity in each clone positively correlated with PD-L1-NL expression levels. To further validate our PD-L1 NanoLuc reporter, HEK293A-PD-L1-NL cells were treated with increasing concentration of GSK3β inhibitor, a well-known degrader of PD-L1 stability [27]. As expected, PD-L1-NL protein levels decreased in a dose-dependent manner, as evidenced by decreased luciferase activity and reduced PD-L1-NL protein levels (Figure 1D,E). These results confirm the successful establishment of a HEK293A cell line stably expressing a NanoLuc-based PD-L1 reporter.

### 2.2. Kinome-Wide High-Throughput Screen (HTS) for Kinase Inhibitors Regulating PD-L1 Stability

**Figure 2:**
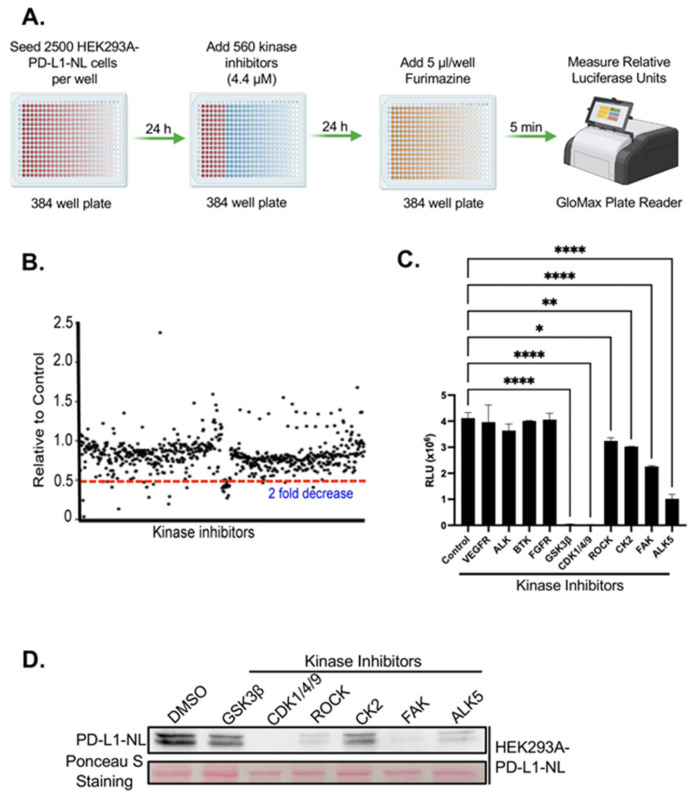
HTS for kinase inhibitors regulating PD-L1 stability. (**A**) Flow diagram of kinome-wide kinase inhibitor screening. (**B**) Results of kinome-wide kinase inhibitors screening. The data represent ratios of relative light units (RLUs) of inhibitor-treated to those of DMSO control. (**C**) Validation of potential kinase inhibitors using luciferase assay. Statistical analysis: *, *p* < 0.05; **, *p* < 0.01; ****, *p* < 0.0001. (**D**) Western blot analysis of PD-L1-NL expression in HEK293A cells. HEK293A-PD-L1-NL cells were treated with 5 µM for 1 day, followed by Western blot analysis. Protein on the membrane was stained with Ponceau S as protein loading control.

Next, we performed a kinome-wide HTS to identify kinase inhibitors that regulate PD-L1 stability. To minimize indirect effects associated with prolonged drug exposure, HEK293A-PD-L1-NL cells were treated with a library of 560 inhibitors targeting the human kinome for 24 h, followed by luciferase assays (Figure 2A). Approximately 19 inhibitors were identified that reduced luciferase activity by more than two-fold (Figure 2B; Table S1). Among these, inhibitors targeting 3 previously known PD-L1 regulators including GSK3β, CK2, ROCK were confirmed [19,20,27], while 16 represented novel candidate regulators of PD-L1 stability. We further validated the screening hits by luciferase assays of 10 out of 16 inhibitors. Of the 10 inhibitors tested, 6 were confirmed to significantly suppress PD-L1-NL luciferase activity (Figure 2C). To determine whether the reduced luciferase signal reflected decreased PD-L1-NL protein levels, we treated HEK293A-PD-L1-NL cells with the validated inhibitors and performed Western blot analysis. All six inhibitors markedly reduced PD-L1-NL protein levels (Figure 2D). Taken together, these results identified six kinases, including three novel kinases: CDK1/4/9, FAK, and ALK5, as regulators of PD-L1 stability.

### 2.3. Validation of FAK as Novel Regulator of PD-L1 Stability

**Figure 3:**
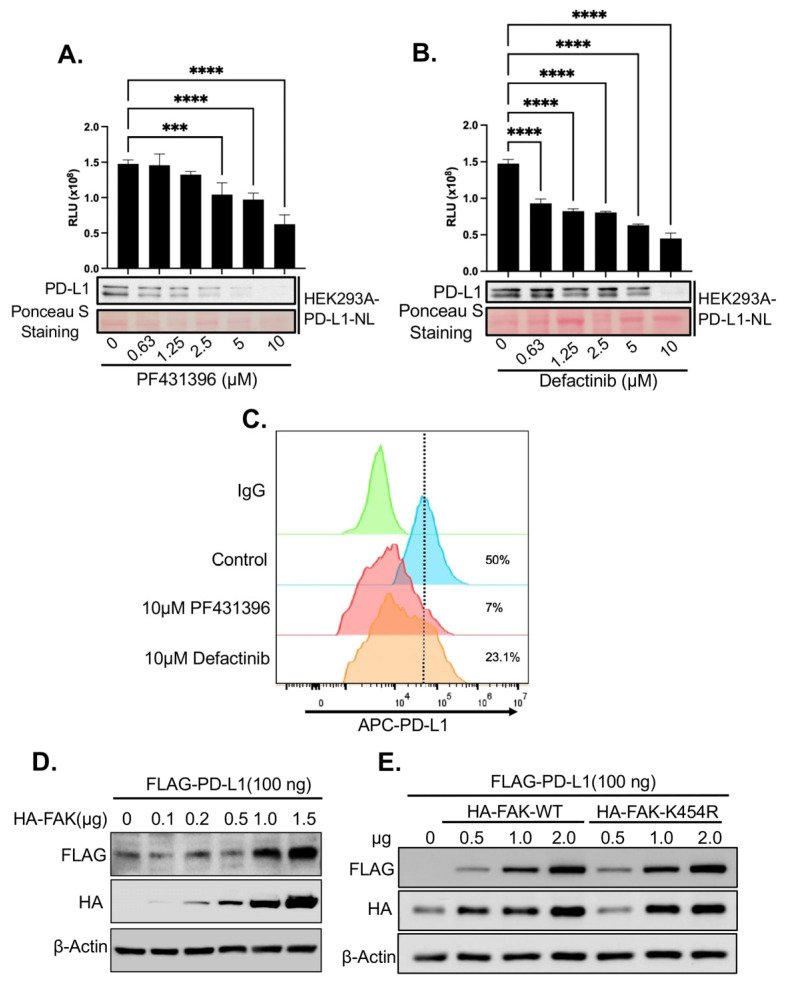
Validation of FAKi-induced PD-L1 degradation in HEK293A cells. (**A**) Dose-dependent effect of FAKi PF431396 on PD-L1 stability. Lower panel: Western blot analysis. Upper panel: luciferase assay. Statistical analysis: ***, *p* < 0.001; ****, *p* < 0.0001. (**B**) Dose-dependent effect of FAKi Defactinib on PD-L1 stability. Lower panel: Western blot analysis. Upper panel: luciferase assay. Statistical analysis: ****, *p* < 0.0001. (**C**) Fluorescence-activated cell sorting (FACS) analysis of the effect of various FAKi on membrane PD-L1. (**D**) Dose-dependent effect of FAK on PD-L1 stability. (**E**) Kinase activity-independent effect of FAK on PD-L1 stability. The dotted line indicates the maximum fluorescent signal from the IgG control.

Given the strong effect of FAK inhibitors on PD-L1 stability observed in our screen (Figure 2D) and the established role of FAK in TNBC [28], we further characterized FAK as a novel regulator of PD-L1. First, we validated that the FAK inhibitor PF431396 used in the HTS reduced PD-L1-NL luciferase activity and protein levels in HEK293A cells in a dose-dependent manner (Figure 3A). To exclude potential off-target effects of PF431396, we also treated HEK293A-PD-L1-NL cells with Defactinib, a more specific FAK inhibitor currently undergoing Phase II clinical trials [29]. Similarly to PF431396, Defactinib decreased PD-L1-NL stability in a dose-dependent fashion (Figure 3B).

Since PD-L1 must localize to the cell membrane to engage PD-1 on immune cells and promote immune evasion, we examined whether FAK inhibition affects membrane-bound PD-L1. Treatment with either PF431396 or Defactinib significantly reduced membrane PD-L1 levels in HEK293A cells (Figure 3C).

We then assessed the impact of FAK overexpression on PD-L1 levels. Co-transfection of increasing amounts of HA-tagged FAK with a fixed amount of FLAG-tagged PD-L1 into HEK293A cells led to a dose-dependent increase in PD-L1 protein levels (Figure 3D). Surprisingly, this effect was independent of FAK activity, as a kinase-dead mutant (FAK-K454R) was equally effective at increasing PD-L1 stability as the wild-type (WT) protein (Figure 3E).

**Figure 4:**
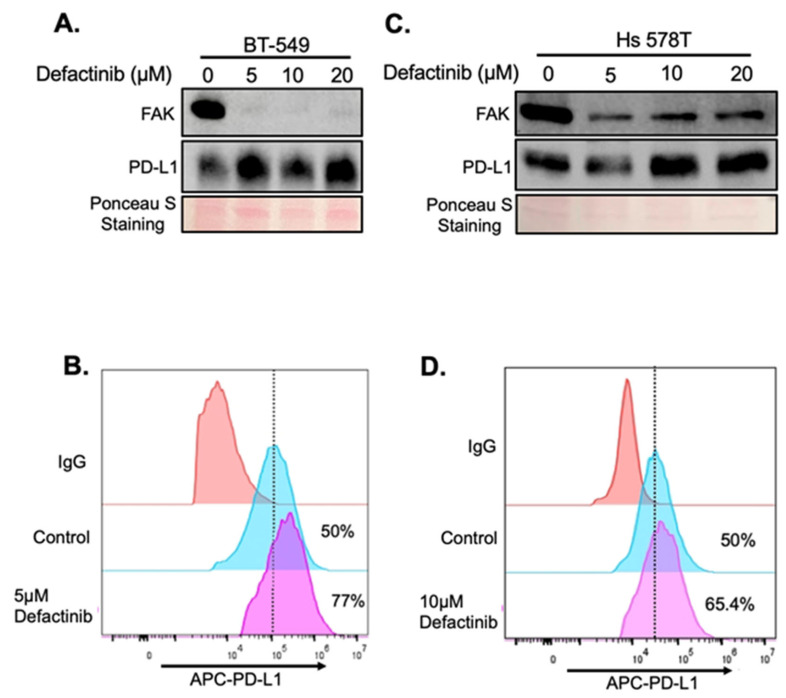
Loss of FAK via inhibition increases PD-L1 stability in BT-549 and Hs 578T cells. (**A**) Western blot analysis of PD-L1. BT-549 cells were treated with 0, 5, 10, and 20 µM of Defactinib for 1 day. Protein on the membrane was stained with Ponceau S Stain. Stained protein was used as loading control. (**B**) FACS analysis of the effect of 5 µM of Defactinib on membrane PD-L1 in BT-549 cells. (**C**) Western blot analysis of PD-L1 in Hs 578T cells were treated with 0, 5, 10, and 20 µM of Defactinib for 1 day. Protein on the membrane was stained with Ponceau S. Ponceau S-stained protein was used as loading control. (**D**) FACS analysis of the effect of 10 µM of Defactinib on membrane PD-L1 in Hs 578T cells. The dotted line indicates the maximum fluorescent signal from the IgG control.

**Figure 5:**
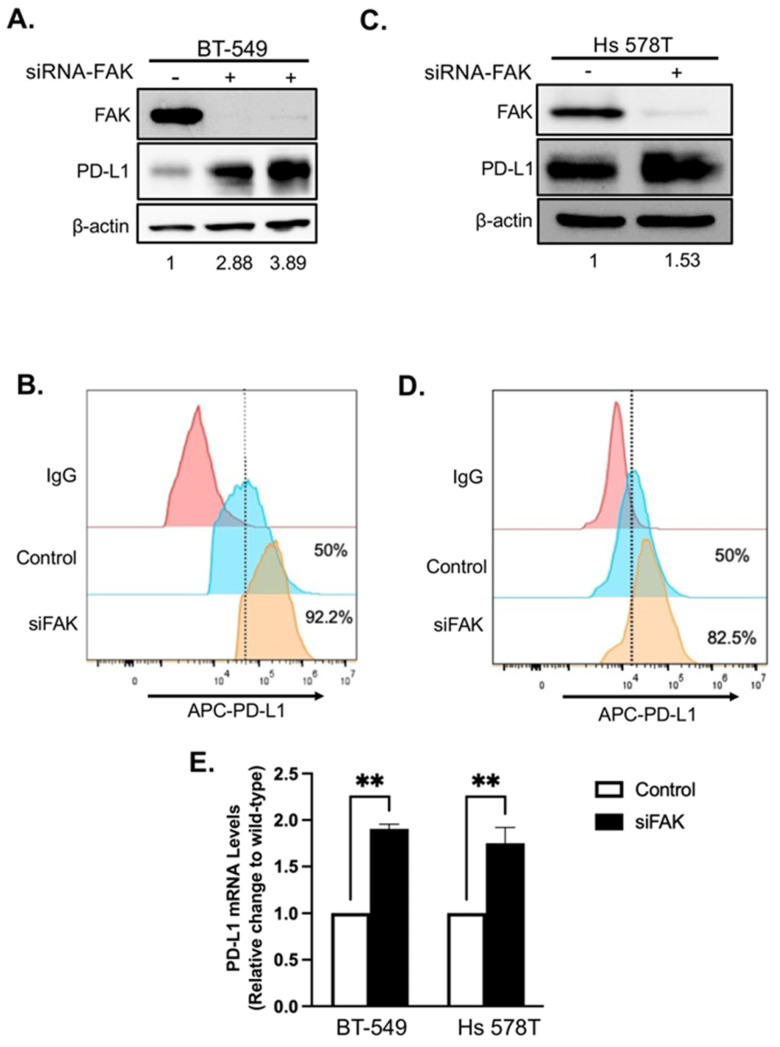
Loss of FAK via siRNA KD increases PD-L1 stability and transcription in BT-549 and Hs 578T cells. (**A**) Western blot analysis of PD-L1 in BT-549 cells after an siRNA-mediated FAK knockdown. β-actin was used as a control. Densitometry calculations were performed using the adjusted band density calculated in ImageLab to compare the PD-L1 expression by dividing the adjusted band density of the siFAK samples by that of β-actin’s adjusted density, resulting in the relative increase in PD-L1 protein expression. (**B**) FACS analysis of the effect of siFAK on membrane PD-L1 in BT-549 cells. (**C**) Western blot analysis of PD-L1 in Hs 578T cells after an siRNA-mediated FAK knockdown. β-actin was used as a control. Densitometry analysis was as described in Figure 5A legend. (**D**) FACS analysis of the effect of siFAK on membrane PD-L1 in Hs 578T cells. (**E**) Quantitative Real-Time qRT-PCR analysis of TNBC cell lines after siRNA-mediated FAK knockdown. Relative change in PD-L1 RNA levels before (Wild-type) and after siFAK-mediated FAK knockdown (siFAK) in BT-549 and Hs 578T cells. The dotted line indicates the maximum fluorescent signal from the IgG control. Statistical analysis: **, *p* < 0.01.

To determine whether FAK also regulates PD-L1 in TNBC cells, we treated two FAK- and PD-L1-positive TNBC cell lines, BT-549 and Hs 578T, with increasing concentrations of Defactinib. Unexpectedly, FAK inhibition in these cells led to elevated levels of both total and membrane-bound PD-L1 (Figure 4A–D), in contrast to the effects observed in HEK293A cells. To confirm that this upregulation was due to specific inhibition of FAK, we performed short interfering RNA (siRNA)-mediated knockdown of FAK in both cell lines. Consistent with Defactinib treatment, FAK knockdown by siRNA (siFAK) significantly increased total and membrane PD-L1 levels in both TNBC cell lines (Figure 5A–E).

Since the PD-L1-NL construct is driven by a CMV promoter, changes in luciferase signal in HEK293A cells reflect post-transcriptional regulation. However, because TNBC cells retained the endogenous PD-L1 promoter, it remained unclear whether the increase in PD-L1 levels following FAK inhibition was due to enhanced transcription. To address this, we measured PD-L1 mRNA levels following FAK knockdown. Interestingly, siFAK also increased PD-L1 mRNA expression in both TNBC cell lines (Figure 5E), suggesting that FAK can regulate PD-L1 at the transcriptional level in these cells as well.

**Figure 6:**
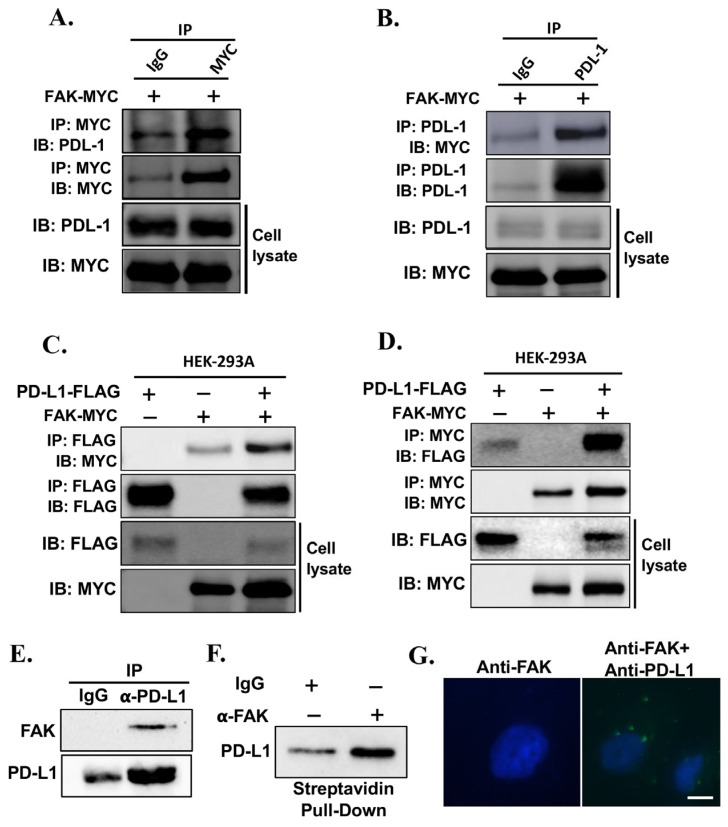
PD-L1 interacts with FAK in vivo. (**A**,**B**) PD-L1 interacts with FAK in vivo. PD-L1 stably overexpressed HEK-293A-pNLF1C-PDL-1 cells were transfected with MYC-tagged-FAK plasmids. The cells were harvested in 1% NP-40 lysis buffer. After checking the expression level of MYC-tagged-FAK or PD-L1, equal amount of cell lysates was subjected to co-immunoprecipitation assays using anti-rabbit-IgG or anti-MYC antibody (**A**) and anti-rabbit or anti-PD-L1 antibodies (**B**) respectively and immunoblotting analysis were performed using anti-PD-L1 or anti-FAK antibody respectively. (**C**,**D**) PD-L1 interacts with FAK in vivo. HEK-293A cells were transfected with MYC-tagged-FAK plasmids or SFB-tagged PD-L1 alone or together. The cells were harvested in 1% NP-40 lysis buffer. After checking the expression level of MYC-tagged-FAK or FALG-PD-L1, equal amount of cell lysates was subjected to co-immunoprecipitation assays using anti-FLAG antibody (**C**) or anti-MYC antibody (**D**) and immunoblotting analysis were performed using anti-MYC or anti-FLAG antibody, respectively. (**E**) Co-IP analysis of FAK-PD-L1 interaction in BT-549 cells. About 1 mg of BT-549 protein lysate was subjected to IP using 2 μg of IgG (control) or rabbit monoclonal anti-PD-L1 antibody. The precipitated proteins were analyzed by Western blot using mouse monoclonal anti-FAK antibody. The membrane was stripped and re-probed with anti-PD-L1 antibody. (**F**) ProtA-TurboID analysis of FAK-PD-L1 in BT-549 cells. Cells were permeabilized and incubated with IgG (control) or anti-FAK antibody in the presence of ProtA-TurboID. FAK-interacting proteins were biotinylated by TurboID and pulled down with streptavidin. Biotinylated PD-L1 was detected by Western blot analysis using anti-PD-L1 antibody. (**G**) PLA analysis of FAK-PD-L1 interaction in BT-549 cells. Cells were cultured on a coverslip and subjected PLA analysis using anti-FAK antibody alone or anti-FAK plus anti-PD-L1 antibody. Nucleus is stained with DAPI (blue). Green fluorescent-dots represent amplified fluorescent probe signal due to FAK-PD-L1 interaction. Scale bar represents 100 μm.

To further investigate whether FAK directly interacts with PD-L1 and regulates its stability, we examined the physical association between the two proteins in cells. We transfected HEK293A-PD-L1-NL cells with MYC-tagged FAK, extracted protein and, and performed co-immunoprecipitation (Co-IP) analysis. Immunoprecipitation using an anti-MYC antibody, but not IgG control, successfully pulled down PD-L1-NL (Figure 6A). Conversely, immunoprecipitation of PD-L1-NL using an anti-PD-L1 antibody pulled down MYC-tagged FAK (Figure 6B). This interaction was further confirmed through co-transfection of FAK-MYC and PD-L1-FLAG into HEK293A cells (Figure 6C,D). To further conform endogenous FAK-PD-L1 interactions in TNBC cells, we performed Co-IP, ProA-TurboID, and proximity ligation assay (PLA) analysis using BT-549 cells. Immunoprecipitation of PD-L1 using anti-PD-L1 antibody, but not IgG control, successfully pulled down FAK (Figure 6E). The ProtA-TurboID method detects protein–protein interactions by using a Protein A–TurboID fusion enzyme that binds antibody-targeted proteins and biotinylates nearby interacting partners in living cells, enabling their subsequent identification by streptavidin-based purification and analysis. As shown in Figure 6F, significantly higher levels of PD-L1 were biotinylated in cells incubated with anti-FAK antibody than IgG control (Figure 6F). PLA detects protein–protein interactions by using pairs of antibodies linked to complementary DNA oligonucleotides that, when bound in close proximity, are ligated and amplified to produce a localized fluorescent signal visible under a fluorescence microscope. As shown in Figure 6G, addition of anti-FAK and anti-PD-L1 antibodies rather than anti-FAK antibody alone showed high fluorescent signals in PLA analysis.

Together, these results strongly support the conclusion that FAK directly binds to PD-L1 and regulates its levels in cancer cells.

### 2.4. Upregulation of PD-L1 After FAK Inhibition Causes Increased Immune Evasion

**Figure 7:**
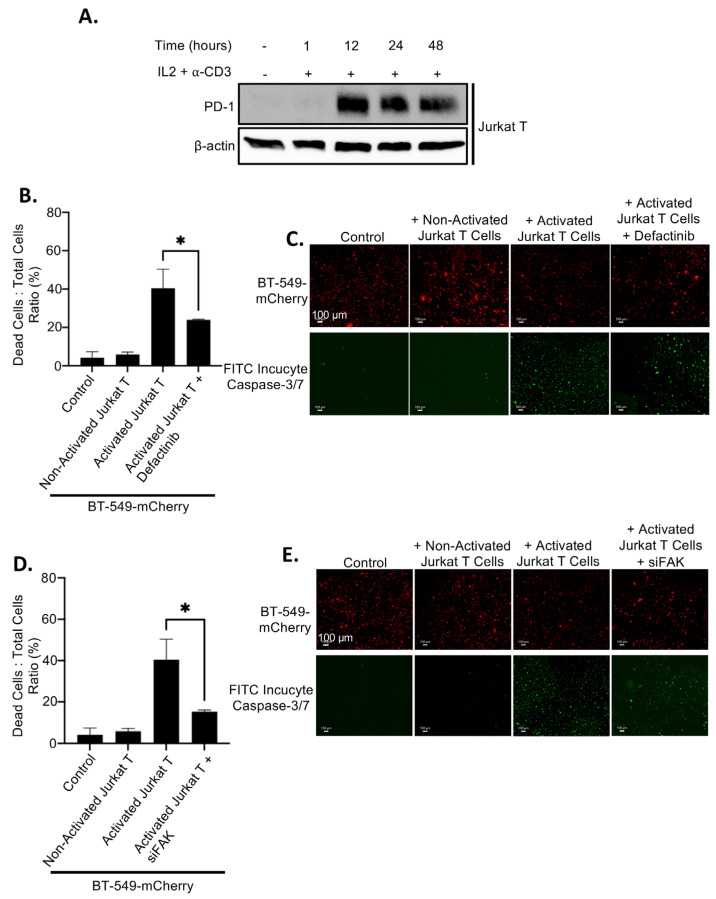
PD-L1 regulation by FAK regulates breast cancer immune evasion. (**A**) Treatment of 20 ng/mL IL-2 and 200 ng/mL of anti-CD3 for 12, 24, and 48 h induces the PD-1 expression in Jurkat T-cells. (**B**) Dead cells: total cells ratio of T-cell killing at in wildtype and 5 µM Defactinib treatment in BT-549-mCherry cells at a 2.5:1 effector to target cell ratio. Statistical analysis: *, *p* < 0.01. (**C**) Fluorescence of live cells (mCherry, red) and dead cells (FITC incucyte caspase–3/7, green) in BT-549-mCherry cells at a 4× magnification. (**D**) Dead cells: total cells ratio of T-cell killing at in wild-type siRNA-mediated FAK knockdown in BT-549-mCherry cells at a 2.5:1 effector to target cell ratio. Statistical analysis: *, *p* < 0.01. (**E**) Fluorescence of live cells (mCherry, red) and dead cells (FITC incucyte caspase–3/7, green) in BT-549-mCherry cells at a 4× magnification.

Since FAK inhibition leads to elevated levels of membrane PD-L1, which may bind to PD-1 on T-cells and promote immune evasion, we assessed whether FAK inhibition affects T-cell-mediated killing of TNBC cells. Jurkat T-cells were activated by treatment with IL2 and anti-CD3 antibody for 12 h, resulting in high PD-1 expression (Figure 7A). As expected, co-culture of inactivated (PD-1-negative) Jurkat T-cells with BT-549 cells had no effect on cancer cell viability. In contrast, co-culture with activated (PD-1-positive) Jurkat T-cells induced substantial cancer cell death (Figure 7B–E). Importantly, inhibition of FAK, either by Defactinib treatment or siFAK, significantly impaired T-cell-mediated killing of BT-549 cancer cells (Figure 7B–E), indicating that FAK inhibition enhances immune evasion by upregulating PD-L1.

## 3. Discussion

TNBC accounts for only 15–20% of all breast cancer cases. However, its high rates of metastasis and recurrence, poor prognosis, and limited treatment options pose significant clinical challenges largely due to the lack of well-defined molecular targets. Therefore, the identification of novel diagnostic and therapeutic targets for TNBC is urgently needed.

FAK is a non-receptor protein tyrosine kinase known to promote tumorigenesis and metastasis by regulating key cancer hallmarks, including cell proliferation, survival, migration, and invasion [29]. Therefore, FAK has emerged as a potential therapeutic target in various cancers. Several small-molecule inhibitors targeting FAK’s kinase function are currently under development and undergoing clinical testing [30]. Despite promising results in preclinical and early-phase trials, no FAK inhibitor has achieved regulatory approval. Notably, FAK is frequently upregulated in TNBC compared with normal breast tissue, supporting it as a therapeutic target in this aggressive subtype [31]. Elevated FAK expression in breast tumors is associated with aggressive features, including increased invasiveness and the triple-negative phenotype, reinforcing its prognostic value and therapeutic relevance [32]. Although FAK inhibitors have shown efficacy in suppressing TNBC cell invasion and tumor growth in preclinical xenograft models [33,34], their clinical application in TNBC has thus far been unsuccessful. The underlying reasons remain unclear. Our findings offer a potential explanation: FAK inhibition increases PD-L1 levels, which may enhance immune evasion in TNBC cells. This mechanism could contribute to the lack of clinical efficacy observed with FAK inhibitors as monotherapy. Therefore, combining FAK inhibitors with immune checkpoint blockade, such as anti-PD-L1 therapy, may represent a more effective strategy for TNBC treatment. Supporting this idea, a recent study reported that the anti-PD-L1 antibody Atezolizumab acts synergistically with FAK inhibition to suppress TNBC cell invasion and motility [33]. Similarly, previous studies have shown that PARP inhibitors (PARPi) upregulate PD-L1 expression and promote immunosuppression. Moreover, combined PARPi and anti-PD-L1 therapy yields superior antitumor effects compared to either agent alone [35]. Together with our findings, these results highlight a broader mechanism whereby targeted therapies may inadvertently promote immune escape through PD-L1 upregulation. Thus, combination strategies that integrate both targeted and immune therapies may provide a promising approach to overcome intrinsic or acquired resistance in TNBC and other cancers.

Our studies identified three novel kinases including FAK, CDK1/4/9, and ALK5 as novel kinase regulators of PD-L1 stability in HEK293A cells after HTS. Further validation also showed that CDK1/4/9 inhibitor but not ALK5 inhibitor significantly reduced PD-L1 levels in TNBC cells (unpublished). It will be very interesting to further explore whether CDK1, CDK4, or CDK9 regulates PD-L1 stability in immune evasion of TNBC cells.

While our discovery of the FAK–PD-L1 signaling axis in immune evasion is novel and promising, several questions remain unanswered. First, the opposing effects of FAK inhibition, decreasing PD-L1 levels in HEK293A cells but increasing them in TNBC cells, are not yet understood. Differential expression of cofactors, phosphatases, or ubiquitin ligases between cell types may account for FAK-mediated PD-L1 regulation. Therefore, the effects of FAK inhibition on PD-L1 stability should be evaluated first when other types of cancer are treated with FAK inhibitors alone and in combination with anti-PD-L1 immunotherapy. Second, although we confirmed a direct interaction between FAK and PD-L1 in cancer cells via co-immunoprecipitation, the mechanistic basis of this interaction remains unclear. PD-L1 is a membrane protein, while FAK is predominantly cytoplasmic. However, FAK has been shown to interact with the intracellular domains of membrane proteins such as integrins and E-cadherin [24], suggesting that FAK may similarly bind the intracellular region of PD-L1. Notably, other kinases known to regulate PD-L1 stability, such as GSK3β, CK2, and ROCK, are also localized in the cytoplasm [20,21,22]. Therefore, it is plausible that FAK interacts with PD-L1 in the cytoplasm during its processing, thereby destabilizing it and preventing membrane localization. In contrast, inhibition of FAK may stabilize PD-L1 and promote its translocation to the membrane. Finally, our current study was conducted entirely in vitro. It remains to be determined whether FAK inhibition promotes immune evasion and tumor progression in vivo. Future studies using immunocompetent syngeneic mouse models, such as 4T1/BALB/c, will be essential to evaluate whether combined treatment with FAK inhibitors and anti-PD-L1 antibodies offers superior efficacy against metastatic TNBC.

## 4. Materials and Methods

### 4.1. Kinase Inhibitors

CHIR99021 (GSK3β inhibitor), SU4312 (VEGFR inhibitor), Asp-3026 (ALK inhibitor), PCI-32765 (BTK inhibitor), BGJ398 (FGFR inhibitor), Y39983 (ROCK inhibitor), P276-00 (CDK1/4/9 inhibitor), PF431396 and Defactinib (FAK inhibitor), and CX-4945 (CK2 inhibitor) were purchased from TargetMol (Wellesley Hills, MA, USA). SJN2511 (ALK5 inhibitor) was purchased from Cayman Chemical (Ann Arbor, MI, USA).

### 4.2. Plasmid Construction

For cloning PD-L1 open reading frame (ORF) into pNLF1-C [CMV/hygro] vector expressing NanoLuc at C-terminus, PD-L1 was amplified by PCR (EcoRI-PD-L1F: 5′-GCTGAATTCACCATGAGGATATTTGCTGTCTT; EcoRV-PD-L1R-no-stop: 5′-ACAGATATCGTCTCCTCCAAATGTGTATC-3′). The PCR product was digested by EcoRI/EcoRV and subsequently cloned into the EcoRI/EcoRV sites of pNLF1-C vector (Promega, Madison, WI, USA) expressing NanoLuc at the C-terminus of protein.

### 4.3. Cell Culture and Establishment of Stable Line

HEK293A (human embryonic kidney cells), HEK293T, and Hs 578T were cultured in Dulbecco’s modified Eagle’s medium (DMEM; Cat# D6429; Sigma-Aldrich, Oakville, ON, Canada). BT-549 was cultured in Roswell Memorial Institute-1640 (RPMI-1640; Sigma R8758). All above-mentioned cells were cultured in medium supplemented with 10% fetal bovine serum, and 1% penicillin/streptomycin (Invitrogen, Burlington, ON, Canada) as antibiotics.

For establishment of a cell line stably expressing PD-L1-NL, HEK293A cells were transiently transfected with PD-L1/pNLF1-C, followed by hygromycin (400 μg/mL) treatment and clonal selection. Individual clones were isolated using cloning rings, trypsinized, and resuspended in DMEM. Cell suspensions were seeded into 96-well plates. Upon confluence, cells were expanded to 12-well plates and analyzed by Western blotting and luciferase assays.

### 4.4. Kinome-Wide Kinase Inhibitor Screen and Drug Validation

Kinome-wide kinase inhibitor screen was performed as described previously [19]. HEK293A-PD-L1-NL cells were used in the screen.

### 4.5. NanoLuc Luciferase Assay

Approximately 1 × 10^4^ HEK293A-pNLF-PD-L1-C20 cells were seeded in triplicate into each well of 96-well plate 24 h prior to treatment. Cells were then treated with increasing concentrations of the indicated drugs. After 24 h, Nano-Glo^®^ Live Cell Reagent (Promega, Madison, WI, USA), containing the cell-permeable furimazine substrate, was added directly to each well. Luminescence was immediately measured using a Promega™ GloMax^®^ Plate Reader.

### 4.6. RNA Extraction and qRT-PCR

RNA extraction, quantification, and qRT-PCR were performed as described [19]. 18S rRNA was used as internal control. The following primers were used in qPCR: rRNA, sense: 5′-TCCCCATGAACGAGGAATTCC-3′, anti-sense: 5′-AACCATCCAATCGGTAGTAGC-3′; PD-L1, sense: 5′-GGCATTTGCTGAACGCAT-3′, anti-sense: 5′-CAATTAGTGCA GCCAGGT-3′.

### 4.7. Knockdown of FAK by siRNA

Knockdown of FAK by siRNA was performed as described previously [36]. siFAKs were purchased from IDT. siFAK sequences are the following: siFAK-1: sense, 5′-AAGCCAACATTGAAUUUCUUCUATC-3′; anti-sense, 5′-GAUAGAAGAAAUUCAAUGUUGGCUUAU-3′; siFAK-2: 5′-AAGACAGTACTTACTATAAAGCTTC-3′; anti-sense: 5′-GAAGCTTTAUAGTAAGTACTGTCTCC-3′.

### 4.8. Immunoblotting, Co-Immunoprecipitation (Co-IP), and Antibodies

Immunoblotting and Co-IP were carried out as described previously [20]. Briefly, cells grown to 70% to 80% confluency were harvested in RIPA/1% NP-40 lysis buffer. Protein samples were subjected to SDS-PAGE and immunoblotted with respective antibodies using standard protocols. β-actin was used as internal control for siRNA KD assays, and the Ponceau S Stain was used for analyses that involved treating cells with kinase inhibitors. Ponceau S was used instead of conventional housekeeping proteins because the use of inhibitors was shown to interfere with the expression of genes such as GAPDH, β-actin, and α-tubulin. Therefore, we decided that using the Ponceau S staining was the best option for a control for inhibitor treatment analyses. Densitometry calculations were performed using the adjusted volume calculated in the ImageLab 6.1 software (Bio-Rad, Mississauga, ON, Canada) and dividing the sample adjusted volume by β-actin’s adjusted volume resulting in the relative increase in PD-L1 protein expression.

For Co-IP, cells expressing different genes were harvested in 1% NP-40 lysis buffer. After checking the expression of different proteins, equal amounts of proteins were precleared overnight at 4 °C using Protein A/G-agarose. The supernatant was subjected to immunoprecipitation using anti-HA-F7 or FLAG-M2 antibodies for 2 h at 4 °C. After the incubation, 20 μL of Protein A/G-agarose was added for an additional 1 h. The beads were washed four times with 150 mM NaCl-1% NP-40 lysis buffer, suspended in 20 μL of 2xSDS sample buffer, boiled for 5 min, and centrifuged. The supernatants were run on SDS-PAGE and blotted with respective antibodies. The antibodies used for Western blot analysis were as follows: PD-L1 (E1L3N, New England Biolabs, Whitby, ON, Canada), anti-FLAG-M2 and β-actin from MilliporeSigma (Etobicoke, ON, Canada); antibodies against HA-F7 and FAK (D-1) from Santa Cruz Biotechnology (Santa Cruz, CA, USA); and mouse monoclonal anti-Myc (9E10) from Roche (Mississauga, ON, Canada).

### 4.9. Fluorescence-Activated Cell Sorting (FACS) Analysis of Membrane PD-L1

FACS analysis of membrane PD-L1 was conducted following a standard protocol provided by Abcam. A PE-conjugated anti-human PD-L1 antibody (clone MIH1, eBioscience) was used for detection, with mouse IgG1 (clone B11/6, Abcam) serving as the isotype control. Briefly, 1 × 10^6^ cells were resuspended in 100 μL of staining buffer containing either the control IgG or APC-conjugated anti-PD-L1 antibody (diluted 1:100) and incubated at room temperature for 30 min on a rotator in the dark. After staining, cells were washed three times with 250 μL of staining buffer. Following centrifugation, the cell pellets were resuspended in 0.5 mL of cell staining Buffer, filtered through a 35 μm cell strainer (VWR), and maintained on ice in the dark until analysis. Flow cytometry was performed using a Beckman Coulter CytoFlex S instrument (Beckman Coulter, Brea, CA, USA).

### 4.10. Immune Evasion Assays In Vitro

To enable co-culture assays, BT-549 cells were labeled with red fluorescence by infection with a lentivirus expressing mCherry. Jurkat T-cells were activated by treatment with anti-CD3 antibody (200 ng/mL) and IL-2 (20 ng/mL) for 12 h to induce PD-1 expression prior to co-culture. 2 × 10^4^ cancer cells were seeded in triplicate into each well of a 96-well plate. After overnight incubation, approximately 2 × 10^6^ activated Jurkat T-cells were added to each well containing cancer cells, followed by the addition of Incucyte^®^ Caspase-3/7 Apoptosis Reagent (Green dye, 5 μM; Sartorius, Oakville, ON, Canada, Cat# 4440). Cancer cell killing was monitored and quantified by fluorescence imaging over 24 h using the CellCyte system (CYTENA, Boston, MA, USA). Red fluorescence indicated live cancer cells, while green fluorescence indicated apoptotic (dead) cells. The percentage of green (dead) cells relative to the total cell population (green + red) was calculated along with standard deviation.

### 4.11. ProtA-TurboID Analysis of Protein–Protein Interaction

Approximately 1.2 × 106 BT-549 cells were permeabilized on a rotator at room temperature for 10 min with 0.04% digitonin buffer (20 mM HEPES KOH pH 7.5, 150 mM NaCl, and 0.5 mM spermidine) before being treated with 1 μg of mouse (G3A1) IgG1 (Cell Signaling Technology, Boston, MA, USA; Cat# 5415, 2.5 mg/mL) or mouse monoclonal anti-FAK antibody (Santa Cruz; H-1: sc-1688) at 600 rpm for 20 min at room temperature. Each sample was then washed three time and resuspended with 0.35 μg of ProtA-TurboID (EMD Millipore Crop., Billerica, MA, USA, PAT001; 0.7 mg/mL) in digitonin buffer. The resuspended mixtures were incubated at 4 °C on the rotator for 30 min and mixed at 600 rpm for 5 min at room temperature. The biotinylating reaction was performed at 1000 rpm at 37 °C for 10 min after washing away the unbound enzyme. The cells were washed once and lysed in 1xRIPA buffer supplemented with protease and phosphatase inhibitors overnight at 4 °C. The supernatant was collected after centrifugation at 13,200 rpm for 10 min at 4 °C. Biotin labeled proteins (PD-L1) were pulled down with 40 μL of streptavidin beads (Cytiva; Marlborough, MA, United Sates; Cat# 28985799) for 30 min at 4 °C. After incubation, the beads were boiled at 100 °C with 20 μL of 2xSDS dye for 5 min. Eluted proteins were subjected to Western Blot analysis using anti-PD-L1 antibody (Cell Signaling Technology, Boston, MA, USA; Cat# 13684).

### 4.12. The Proximity Ligation Assay (PLA) Analysis of Protein–Protein Interaction

The Duolink^®^ Proximity Ligation Assay (PLA) was performed in BT549 cells to detect protein–protein interactions between PD-L1 and FAK. Cells were fixed with 4% paraformaldehyde, permeabilized with 0.2% Triton X-100/1×PBS and incubated with primary antibodies against PD-L1 (rabbit) and FAK (mouse). Species-specific PLA probes (Anti-mouse PLUS and Anti-rabbit MINUS; MilliporeSigma, Etobicoke, ON, Canada) were added, followed by ligation and rolling-circle amplification using Duolink^®^ Fluorescent Detection Reagent (MilliporeSigma), generating fluorescent signals at interaction sites. Samples were counterstained with DAPI and mounted for microscopy. Negative controls included anti-FAK alone. PLA signals appeared as discrete fluorescent spots, visualized by fluorescence microscopy, allowing visualization of PD-L1–FAK interactions relative to negative controls.

### 4.13. Statistical Analysis

Statistical analyses were performed using the Prism 10 program.

## Data Availability

The original contributions presented in this study are included in the article/Supplementary Materials. Further inquiries can be directed to the corresponding author.

## Supplementary Materials

The supporting information can be downloaded at: https://www.mdpi.com/article/10.3390/ijms262010108/s1.

## Abbreviations

The following abbreviations are used in this manuscript:

AKT	Protein Kinase B
ALK	Anaplastic Lymphoma Kinase
APC	Allophycocyanin (fluorescent dye)
BTK	Bruton’s Tyrosine Kinase
CDK	Cyclin-Dependent Kinase
CK2	Casein Kinase 2
Co-IP	Co-immunoprecipitation
DMEM	Dulbecco’s Modified Eagle Medium
FACS	Fluorescence-Activated Cell Sorting
FAK	Focal Adhesion Kinase
FDA	U.S. Food and Drug Administration
FGFR	Fibroblast Growth Factor Receptor
GSK3β	Glycogen Synthase Kinase 3 Beta
HA	Hemagglutinin (epitope tag)
HEK293A	Human Embryonic Kidney 293A cells
HTS	High-Throughput Screening
ICI	Immune Checkpoint Inhibitor
IL2	Interleukin 2
MAPK	Mitogen-Activated Protein Kinase
NL	NanoLuc
NP-40	Nonidet P-40 (detergent)
NanoLuc	NanoLuciferase
ORF	Open Reading Frame
PBS	Phosphate-Buffered Saline
PD-1	Programmed Cell Death Protein 1
PD-L1	Programmed Death-Ligand 1
PDX	Patient-Derived Xenograft
PE	Phycoerythrin (fluorescent dye)
PI3K	Phosphoinositide 3-Kinase
RIPA	Radioimmunoprecipitation Assay buffer
ROCK	Rho-associated Protein Kinase
TAZ	Transcriptional coactivator with PDZ-binding motif
TNBC	Triple-Negative Breast Cancer
VEGFR	Vascular Endothelial Growth Factor Receptor
WT	Wild-Type
mTOR	Mechanistic Target of Rapamycin
qRT-PCR	Quantitative Real-Time PCR
siFAK	siRNA targeting FAK
siRNA	Small Interfering RNA
