# Supplementary material for: Kinome-Wide Screening Identifies FAK as a Novel Post-Translational Regulator of PD-L1 Stability and Immune Evasion in Triple-Negative Breast Cancer
Source: Int J Mol Sci. 2025 Oct 17;26(20):10108. doi: 10.3390/ijms262010108 (PMC12562877; doi:10.3390/ijms262010108)
Supplement: Supplementary file 1 [file ijms-26-10108-s001.zip › ijms-3825202-supplementary.pdf]

Table S1. Kinase Inhibitors Reducing PD-L1-NL Luciferase Activity More Than 2-fold

No.	**Kinase Inhibitor Names**	**Kinase**	**Fold change**
1	CHIR-99021 , CT-99021,	GSK	0.035
2	Lapatinib ditosylate, Tykerb, GW572016	EGFR/HER2	0.133
3	GW583340 dihydrochloride, GW-583340	EGFR//HER2	0.195
4	CHIR-98014 isomer, CT-98014	GSK3β	0.242
5	JNJ 28871063 hydrochloride	EGFR, HER2/4	0.281
6	GW8510, GW-8510	EGFR/HER2	0.290
7	P276-00, Riviciclib hydrochloride	CDK1/4/9	0.300
8	GSK-1059615, GSK-615	PI3K/mTOR	0.305
9	ASP-3026	ALK	0.351
10	GLPG-0259, Compound A	MAPKAPK5<br>MK5, PRAK	0.361
11	PF 3644022	MAPKAPK2	0.400
12	Y39983, RKI-983, SNJ-1656	ROCK	0.400
13	CX-4945, Silmitasertib	CK2	0.400
14	SJN 2511	ALK5	0.405
15	NVP-BHG712, NVP-BHG-712	Eph4	0.408
16	BGJ-398,	FGFR	0.409
17	AG13958, AG-013958	VEGFR	0.411
18	PCI-32765, Ibrutinib	BTK	0.423
19	PF431396	FAK	0.445
